# Epidemiological Interface of Sylvatic and Dog Rabies in the North West Province of South Africa

**DOI:** 10.3390/tropicalmed7060090

**Published:** 2022-06-05

**Authors:** Ayla J. Malan, Andre Coetzer, Claude T. Sabeta, Louis H. Nel

**Affiliations:** 1 Department of Biochemistry, Genetics and Microbiology, Faculty of Natural and Agricultural Sciences, University of Pretoria, Pretoria 0002, South Africa; u14390257@tuks.co.za (A.J.M.); andre.coetzer@rabiesalliance.org (A.C.); 2 Global Alliance for Rabies Control South Africa Non-Profit Company, Pretoria 0181, South Africa; 3 Agricultural Research Council—Onderstepoort Veterinary Institute, World Organisation for Animal Health (OIE) Rabies Reference Laboratory, Onderstepoort, Pretoria 0110, South Africa; claude.sabeta@up.ac.za

**Keywords:** rabies, dog, sylvatic, molecular epidemiology, South Africa

## Abstract

Rabies is a viral zoonosis that causes an estimated 59,000 preventable human fatalities every year. While more than 120 countries remain endemic for dog-mediated rabies, the burden is the highest in Africa and Asia where 99% of human rabies cases are caused by domestic dogs. One such rabies-endemic country is South Africa where an estimated 42 preventable human deaths occur every year. Although canine rabies had been well described for most of the provinces in South Africa, the epidemiology of rabies within the North West Province had not been well defined prior to this investigation. As such, the aim of this study was to use nucleotide sequence analyses to characterise the extant molecular epidemiology of rabies in the North West Province of South Africa—with specific focus on the interface between dogs and sylvatic species. To this end, *Rabies lyssavirus* isolates originating from the North West Province were subjected to molecular epidemiological analyses relying on the Bayesian Markov Chain Monte Carlo methodology on two distinct gene regions, viz. the G-L intergenic region and partial nucleoprotein gene. Our results provided strong evidence in support of an endemic cycle of canine rabies in the East of the province, and three independent endemic cycles of sylvatic rabies spread throughout the province. Furthermore, evidence of specific events of virus spill-over between co-habiting sylvatic species and domestic dogs was found. These results suggest that the elimination of canine-mediated rabies from the province will rely not only on eliminating the disease from the dog populations, but also from the co-habiting sylvatic populations using oral rabies vaccination campaigns.

## 1. Introduction

Rabies is a viral zoonosis caused by various members of the *Lyssavirus* genus belonging to the *Rhabdoviridae* family in the order *Mononegavirales* [1]. While all the species in the *Lyssavirus* genus are causative agents for the disease rabies, the prototype member is *Rabies lyssavirus* (RABV), which has the greatest public health impact due to its association with domestic dogs (*Canis lupus familiaris*) [2]. While canine-mediated rabies has been eliminated from some regions and territories around the world [3,4], the disease is still endemic to every landmass except for Antarctica and a few isolated islands [2].

South Africa—a rabies-endemic country in southern Africa—has made considerable progress towards eliminating canine-mediated rabies [5], but it is estimated that approximately 42 canine-mediated human rabies cases still occur every year [6]. While records suggest that rabies may have been present in South Africa for more than a century [7], six of the nine South African provinces (viz. Limpopo (LP), North West (NW), Mpumalanga (MP), KwaZulu-Natal (KZN), Eastern Cape (EC), and Free State (FS) provinces) are currently considered endemic for canine-mediated rabies. In contrast, the three remaining provinces (Northern Cape (NC), Western Cape (WC), and Gauteng (GP) provinces) have been shown to only experience sporadic cases and outbreaks and are thus considered vulnerable to outbreaks but not endemic to canine-mediated rabies [8,9,10,11,12,13].

In addition to canine-mediated rabies, the epidemiology of rabies in South Africa is further complicated by the occurrence and geographical distribution of various sylvatic reservoir species that can maintain and transmit rabies. In evidence of the fact, jackal species (black-backed jackal, *Canis mesomelas,* and side-striped jackal, *Canis adustus*) and bat-eared foxes (*Otocyon megalotis*) have been shown to be capable of maintaining the canine variant of the RABV in South Africa [9,14], while members of the *Herpestidae* family (e.g., the yellow mongoose (*Cynictis penicillata*)) can transmit the mongoose variant of RABV [15,16]. While jackal populations are most prominently found in subsistence and commercial farming areas, as well as bushveld areas, they appear to be the dominant maintenance host in the northern areas of South Africa [14,17,18]. Despite having a relatively large geographical distribution throughout South Africa, bat-eared fox populations appear to be the dominant maintenance host in the western areas of the country [17]. In contrast, rabies cases in mongoose populations (capable of transmitting the mongoose variant of the RABV) are endemic to the central plateau of South Africa [16].

Evidence from previous studies from South Africa and elsewhere suggests that rabies-positive sylvatic species pose a negligible public health impact due to the limited interaction between humans and wildlife in general [8,19]. However, sylvatic reservoir species still pose a risk of reintroducing the disease into immunologically naïve domestic dog populations where ecological niches overlap—highlighting the importance of understanding the epidemiology and transmission dynamics of endemic cycles of sylvatic rabies [20].

To this end, molecular epidemiological investigations have been implemented extensively in South Africa to gain an improved understanding of rabies within a particular area and to investigate the interface between canine and sylvatic rabies. In evidence, the epidemiology of RABV in the LP [8,14,21], MP [14], GP [12], KZN [13,22,23,24], WC [9,25], FS [10], and the NC [7,9,26] provinces had been investigated to gain insights into rabies and its transmission dynamics in the country.

None of the studies undertaken to-date had, however, focussed specifically on the NW Province, which is situated in the northern regions of South Africa where it shares political borders with four South African provinces (viz. NC, FS, GP, and LP provinces) and the neighbouring country of Botswana. While the first case of canine-mediated rabies in the NW province was only identified in 1980, published findings suggest that rabies was introduced into the province in the 1950s from the neighbouring LP Province (formerly part of the Transvaal province) after spreading from either Botswana or Zimbabwe [7]. In addition to being endemic for dog-transmitted rabies, historical surveillance data suggested that sylvatic carnivore species such as jackals within the province were also routinely diagnosed as rabies-positive, suggesting that the sylvatic populations were able to maintain disease transmission and thus contribute to the persistence of rabies [17].

As no prior epidemiological studies had focused on the NW Province, the relationship between domestic and wildlife rabies cycles in the province were not particularly well defined. As such, the aim of this study was to establish and evaluate the genetic relationships between RABV sequences obtained from sylvatic and canine species within the NW Province of South Africa through a molecular epidemiological analysis of both the G-L intergenic regions and the nucleoprotein genes (N). In the next step, this cohort of NW virus sequences were also compared with known virus sequences published for the larger geographical region, including a number of additional South African provinces and other southern African countries. The findings in this study demonstrated the circulation of different closely linked RABV clusters and identified specific RABV cycles where sylvatic species were the primary maintenance hosts.

## 2. Materials and Methods

### 2.1. Sample Cohort Used in the Study

A cohort of samples was selected based on the species and the geographical distribution throughout the NW Province. These RABV-positive samples (*n* = 51) were collected as part of South Africa’s routine surveillance work between 2017 and 2019 (subjected to rabies confirmatory testing by means of direct fluorescent antibody (DFA) test at the Agricultural Research Council—Onderstepoort Veterinary Institute (ARC-OVI, Pretoria, South Africa)). The animal species from which samples originated were: canine (*n* = 11); bovine (*n* = 20); black-backed jackal (*n* = 9); bat-eared fox (*n* = 1); ovine (*n* = 2); caprine (*n* = 3); unidentified jackal species (*n* = 3); aardwolf (*Proteles cristata*) (*n* = 1); and genet (*Genetta genetta*) (*n* = 1).

### 2.2. Total RNA Extraction

Total RNA was extracted from RABV-positive brain samples (*n* = 51) by using the Zymo Direct-zol RNA MiniPrep Plus kit (Zymo Research, Irvine, CA, USA) according to the manufacturer’s instructions.

### 2.3. Reverse Transcription and Amplification of the Glycoprotein Gene and the Adjacent G-L Intergenic Region

Viral genomic cDNA was synthesised [27] and the cytoplasmic domain of the glycoprotein gene and the adjacent G-L intergenic region was amplified using the G(+) and L(-) primer sets [28,29]. PCR amplicons (*n* = 51) were electrophoresed and recovered using the Zymoclean Gel DNA Recovery kit according to the manufacturer’s instructions (Zymo Research).

### 2.4. Reverse Transcription and Amplification of the Partial Nucleoprotein Gene Region

Viral cDNA was synthesised using an initial 1:10 RNA dilution [27]. Thereafter, the partial N gene PCR reaction was set up using 5 μL of cDNA following Taguchi optimisation [30]. The amplification relied on the following cycling conditions: 1 cycle at 94 °C for one minute, 40 cycles at 94 °C for 30 s, 45 °C for 30 s and 72 °C for 90 s, and a final extension cycle at 72 °C for seven minutes. The amplified PCR-positive products (*n* = 51) were electrophoresed and purified as mentioned previously (Zymo Research).

### 2.5. Sanger Sequencing

The forward and reverse sequences for both amplified gene regions (G-L intergenic region and partial N gene) were sequenced at Inqaba Biotec™ (Pretoria, South Africa) using the ABI Prism 3500XL Genetic Analyzer (ThermoFisher, Waltham, MA, USA). The final consensus sequences were trimmed to 405 nucleotides (nt) for the partial N gene and 592 nt for the G-L intergenic region using the CLC Main Workbench software (version 20.0.1). The trimmed consensus sequences—representing the G-L intergenic region and partial N gene of the RABV genome for samples from the NW (*n* = 51) province—were submitted to the NCBI GenBank and allocated unique accession numbers (G-L: MW343859–MW343912; N: MW343969–MW344022; Table S1).

### 2.6. Phylogenetic Analyses

The phylogenetic analysis included the 51 RABV sequences from the NW province as well as published G-L intergenic region (*n* = 22) and partial nucleoprotein gene (*n* = 25) sequences that had been obtained from South Africa (NW, GP, LP, FS, and KZN provinces) and the neighbouring countries of Zimbabwe and Botswana (Table S1).

Two sequence alignments were created viz. for the partial N gene sequences (*n* = 77) and for the G-L intergenic region sequences (*n* = 76). The sequences for both datasets were aligned using the ClustalW subroutine of the MEGA X software package [31], and the best-fitting DNA substitution models (partial N gene: TrN+G; G-L intergenic region: TIM1+G) were determined using the JModel software package (version 2.1.10) using the Akaike’s information criterion (AIC).

The final phylogenetic analysis for both gene regions was undertaken using a Bayesian Markov Chain Monte Carlo (MCMC) method in the BEAST software package (version 2.6.0) [32]. The posterior distributions were subsequently inspected using the Tracer software (version 1.7.1) to ensure adequate mixing and convergence before the associated statistics were summarised as a maximum clade credibility tree and visualised using the FigTree software (version 1.4.4).

## 3. Results

### 3.1. Molecular Epidemiology of RABVs from the North West Province of South Africa

#### 3.1.1. Based on Partial Nucleoprotein Gene Region

**Figure 1:**
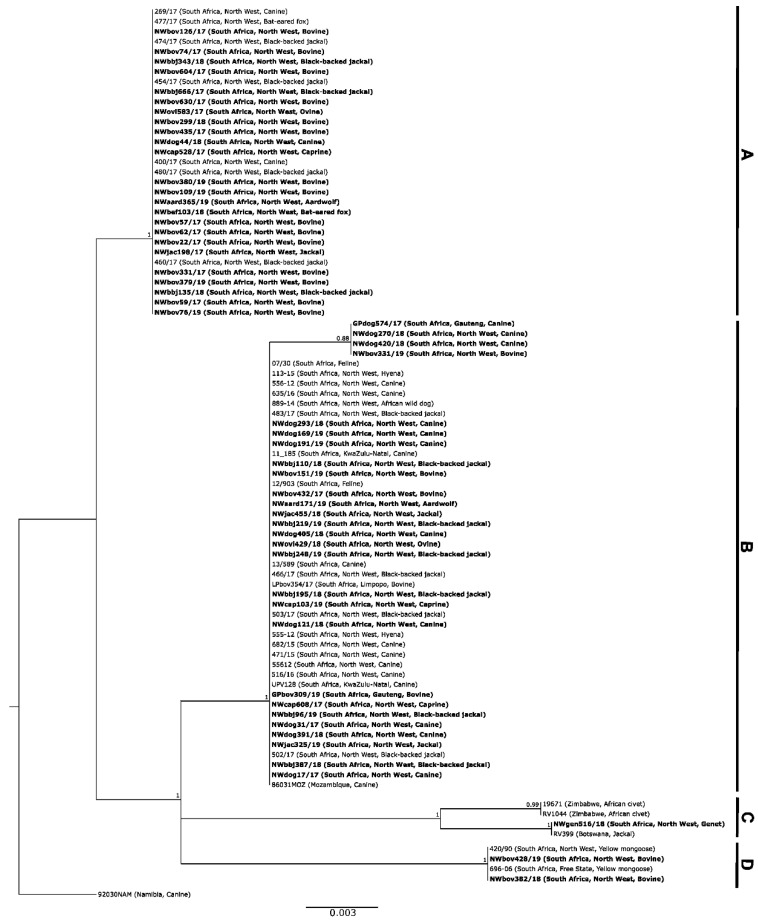
Maximum clade credibility tree for partial N gene sequences derived from South Africa, Zimbabwe, and Botswana (Table S1). The horizontal branch lengths are proportional to the homology between sequences within and between groups and all branches with a posterior probability of ≤0.75 were collapsed. A canine sequence from Namibia (92030NAM) was used to root the tree. The new sequences generated in this study are shown in bold (Table S1). All sequences in Clade A and Clade B belong to the Africa 1-b lineage, while the sequences in Clade C and Clade D belong to the Africa 3 lineage (Table S1).

**Figure 2:**
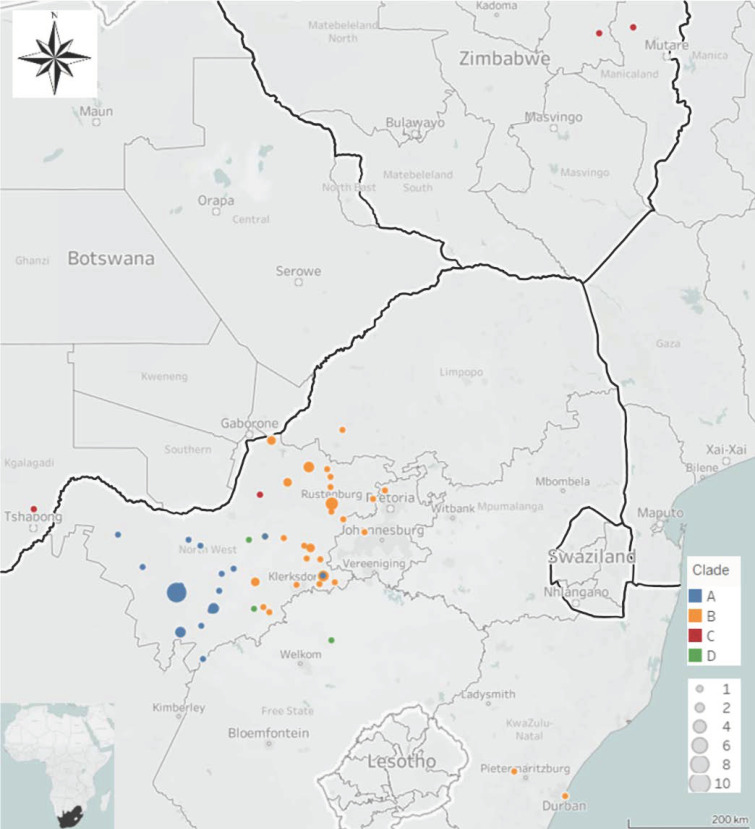
Geographic distribution for RABV sequences from each clade as seen in the phylogenetic analysis for the partial N gene and G-L intergenic region sequences.

The partial N gene sequences disaggregated into four separate clades (A–D) with each clade supported by high posterior probabilities (Figure 1). Clade A consisted of 30 RABV sequences that originated from various host species all found in the western parts of the NW Province (Figure 2). These hosts included canine (*n* = 3), bovine (*n* = 15), black-backed jackal (*n* = 7), an ovine, a caprine, bat-eared fox (*n* = 2), and one unspecified jackal species.

Clade B consisted of 39 RABV sequences from the eastern parts of the NW province (as opposed to those in clade A above) and the neighbouring provinces of GP and LP, as well as the more distant KZN province of South Africa (Figure 1 and Figure 2). In terms of host species, these RABV sequences were collected from canine (*n* = 17), bovine (*n* = 5), black-backed jackal (*n* = 10), caprine (*n* = 2), an ovine, an aardwolf, an African wild dog, and unspecified jackal species (*n* = 2).

Clade C consisted of one RABV sequence from the NW province, one RABV sequence from neighbouring Botswana, and two from Zimbabwe (Figure 2). These sequences were all from wildlife viz. African civet (Zimbabwe), genet (NW, South Africa) and jackal (Botswana).

Finally, Clade D consisted of RABV sequences from southern/central locations in the NW province and the adjacent FS province to the South (Figure 1 and Figure 2). The RABV sequences within this clade were collected from a mongoose from the FS (*n* = 1) and NW provinces (*n* = 1), and bovine samples from the NW Province (*n* = 2). These RABV sequences clustered with known sequences from the mongoose variant, suggesting that the mongoose variant of RABV extended from the neighbouring FS Province into the central parts of the NW Province (Figure 1 and Figure 2).

#### 3.1.2. Based on the G-L Intergenic Region

**Figure 3:**
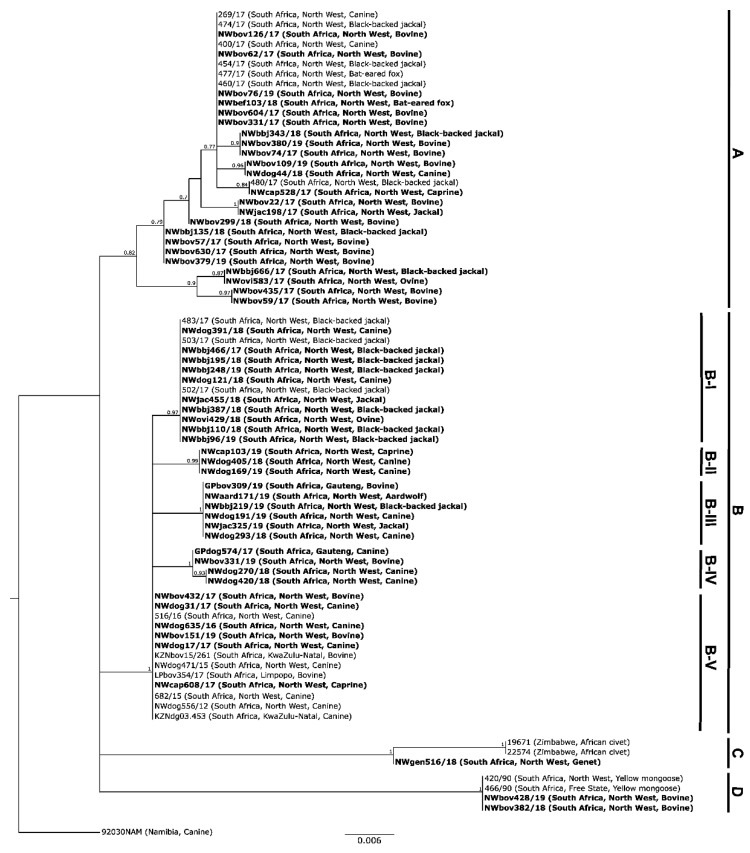
Maximum clade credibility phylogenetic tree for the cytoplasmic domain and G-L intergenic region sequences sourced from samples in South Africa and Zimbabwe (Table S1). The horizontal branch lengths are proportional to the homology between sequences within and between groups and all branches with a posterior probability of ≤0.75 were collapsed. A canine sequence from Namibia (92030NAM) was used to root the tree. The new sequences generated in this study are shown in bold (Table S1). All sequences in Clades A and B belong to the Africa 1-b lineage, while the sequences in Clades C and D belong to the Africa 3 lineage (Table S1).

Phylogenetically, the RABV sequences included in the molecular epidemiological investigation of the G-L intergenic region aggregated into the same clusters demonstrated for the partial N gene sequences, viz. four distinct Clades A to D, each supported by high posterior probabilities (Figure 2 and Figure 3). In the case of Clades A, C, and D, the two genomic regions delivered an identical phylogenetic interpretation for all the sequences included in both analyses.

**Figure 4:**
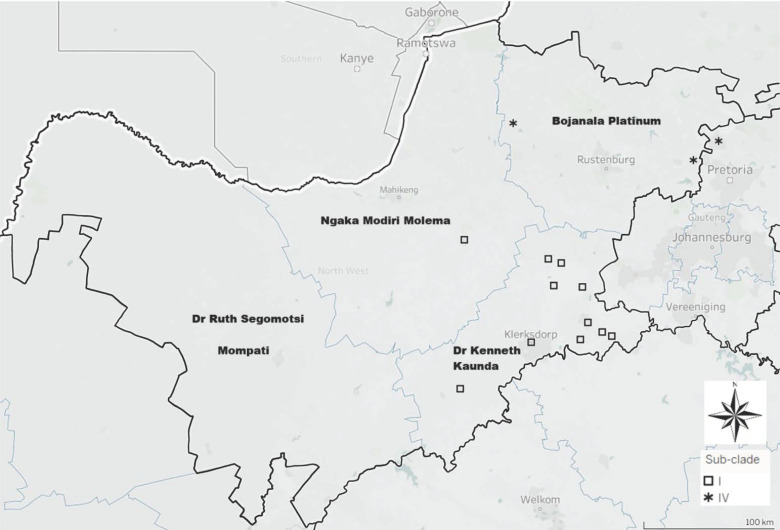
Geographic distribution for each the RABV sequences in sub-clades B-I and B-IV of Clade B.

For Clade B, five additional sub-clades (B-I to B-V) could be observed. This additional resolution could be ascribed to the higher heterogeneity of the G-L intergenic region as compared to the N gene. Sub-clade B-I was geographically associated with the southern parts of the province (Figure 4) and consisted of RABV sequences originating from black-backed jackals (*n* = 9), canine (*n* = 2), an ovine, and an unspecified jackal species. Sub-clade B-IV was geographically associated with the northern regions of the province (Figure 4) and consisted of RABV sequences originating from canine (*n* = 3), and a bovine host species. Sub-clade B-V consisted of RABV sequences originating from bovine (*n* = 4), canine (*n* = 8), and one caprine species. Geographically, occurrence of these cases was widespread and across multiple provinces, viz. LP, NW, and KZN provinces (Figure 2).

The geographical grouping of the remainder of the sub-clades (B-II, B-III) was more indistinct (compared to the above) and overlapped throughout the eastern half of the NW Province (Figure 3). Sub-clade B-II consisted of RABV sequences collected from a caprine and canine (*n* = 2) sample that were from the parts of the NW Province bordering the GP Province. Sub-clade B-III consisted of RABV sequences collected from a bovine, an aardwolf, a black-backed jackal, canine (*n* = 2), and an unspecified jackal species.

## 4. Discussion

Over recent years, rabies has become an increasing concern in the NW province of South Africa, given the apparent expanding involvement of wildlife in the country [9,14,33]. However, very little is known about the epidemiological drivers nor the interplay between dog and wildlife rabies. This study was, however, able to contribute some new information on virus–host interactions and the molecular epidemiology of rabies in this province and the larger geographical region.

The RABV sequences from the NW province formed part of either the Africa 1b sub-lineage that is predominantly found in East Africa [34,35] or the Africa 3 lineage which is largely limited to infection of species of the *Herpestidae* family in southern Africa.

At a more localized level, the RABV sequences included in this study could phylogenetically be divided into four clades (Clades A–D), while the same branching cluster could be observed for both the G-L intergenic region and the partial N gene region of the RABVs. It is evident that the phylogenetic analyses for both the partial N gene and the G-L intergenic region led to similar conclusions, although the phylogeny for the G-L intergenic region provided greater resolution at a localised level.

Clade A consisted almost entirely of RABV sequences collected from sylvatic (~33%) and livestock species (~57%) and clustered independently from all other RABV sequences collected and included from elsewhere in South Africa or any of the neighbouring countries. This finding suggested that a distinct endemic cycle of sylvatic rabies had become established in the western parts of the NW Province. In consideration, it could be noted that ~42% of this part of the NW province was associated with farming, in particular livestock farming [36]. This finding provides support to the notion that the incidence of sylvatic rabies cases may be considerably higher in areas where extensive commercial and subsistence farming are associated with (1) low density of domestic dogs and (2) high jackal population densities—high enough to sustain an intra-species RABV cycle in jackals, with regular spill-over to livestock and occasional transmission to dogs [18,37].

The RABV sequences that formed part of Clade B were collected primarily from rabies-positive dogs from various provinces in South Africa (viz. the NW, GP, KZN, and LP provinces). This finding suggested that a large endemic cycle of canine-mediated rabies was present in the eastern parts of the NW Province, and that this cycle was also linked with other canine RABV cycles that were geographically distinct. To improve the resolution and, in so doing, better define the broader endemic cycle, the observed clade was broken down into distinct sub-clades in the phylogenetic analysis of the G-L intergenic region. Five sub-clades were identified of which two (sub-clades B-I and B-III) consisted primarily of sylvatic rabies cases that had originated from within the Dr Kenneth Kaunda and Bojanala districts in the eastern parts of the NW Province and were thus considered indicative of two endemic cycles of sylvatic rabies. The remaining sub-clades (sub-clades B-II, B-IV, and B-V) consisted primarily of RABV sequences derived from canine and livestock samples from various regions in South Africa. Sub-clade B-II was composed of canine and caprine samples that originated from an area that included the eastern parts of the NW Province (bordering the GP Province) through the south-western regions of the Ngaka Modiri Molema district. This area covers land associated with intensive goat farming [38] and evidently rabid dogs are responsible for the significant incidence of rabies in this livestock species (as opposed to the role and involvement of jackals described for the subclades B-I and B-III). For subclade B-IV, a strong association between rabies cases in livestock (cattle) and a dog rabies cycle was also observed.

Sub-clade B-V on the other hand predominantly involved carnivores only (both dogs and jackals) and covered a large territory (NW, LP, GP, and KZN provinces). Thus, endemic cycles of canine rabies from the NW could be linked to endemic rabies cycles in multiple provinces of South Africa, including the GP Province where an outbreak of canine rabies occurred in 2010 [12]. Prior to 2010, sylvatic rabies cases in GP were predominantly found in the outlying rural areas of the province, and these cases could be linked to the movement of infected individuals between the NW and GP provinces [12]. These findings suggested that the long-range movement of infected animals between provinces in South Africa is far more widespread than originally thought [10,11,12,21,24,39].

Clade C indicated that there was genetic homology between the RABV sequences collected from civet species in the western parts of Zimbabwe, jackal species in the southern parts of Botswana, and a genet in the northern parts of the NW Province. During a previous investigation of the molecular epidemiology of rabies in Zimbabwe, the researchers found strong evidence that a sylvatic cycle of rabies was being maintained by the civet populations in Zimbabwe [33]. During that study, the authors noted that the RABV cycle within the civet populations was related to an outbreak of mongoose rabies circulating within the slender mongoose (*Galerella sanguinea*) [33]. This is consistent with our finding as the civet, genet, and jackal samples in the phylogenetic analyses were distantly related to the canid variant of RABV circulating within the other samples included for study in our analyses. This would suggest that the sylvatic cycle was not only limited to the civet populations of Zimbabwe, but also extended across Zimbabwe into South Africa and Botswana. This observation is, however, speculative as there were no additional RABV sequences from those specific locations in South Africa and Botswana to include in our investigation. As a result, it was not possible to determine whether the jackals in Botswana and genets in South Africa were maintaining the endemic cycle or were incidental hosts that became infected.

Clade D consisted solely of sequences of the mongoose variant of the RABV that had been collected from both mongoose and bovine species originating from the NW and FS provinces. Geographically these territories are consistent with the natural home-range of yellow mongoose species [40], but this is the first report of the presence of the mongoose variant of the RABV in the NW Province.

The RABV endemic cycles circulating within the NW Province could be phylogenetically linked to various cycles circulating within other territories in South Africa (viz. LP, GP, FS, and KZN provinces) and neighbouring countries (Zimbabwe and Botswana). In addition, the results presented in this study also provided strong evidence in support of the establishment of at least three endemic cycles of sylvatic rabies across different parts of the NW Province, where one of these sylvatic cycles was restricted to the western parts of the NW Province. The remaining two sylvatic rabies cycles could be found in the eastern regions of NW Province where the distribution of sylvatic and canine rabies cycles was found to overlap. Despite this, the results presented here show that the sylvatic species within the eastern part of the province were able to maintain rabies independently from dogs in the same geographical area, and both species groups could be implicated in the persistent transmission of RABV to livestock in these regions—potentially impacting future rabies control strategies. Lastly, it could be speculated that a fourth endemic cycle of sylvatic rabies, which appeared to extend between Zimbabwe, Botswana, and South Africa might also have become established in the northern parts of the NW Province.

## 5. Conclusions

While South Africa has made progress towards freedom from canine-mediated human rabies [5], the presence of various sylvatic species capable of maintaining the RABV in geographical areas that overlap with dog populations could hinder future control and elimination programs. In support of this, the findings presented here—and from a previous investigation [9]—suggested that the presence of sylvatic species capable of maintaining RABV transmission is likely to hamper canine rabies elimination. The persistence of sylvatic rabies and the recurring spill-over infections to co-habiting dog populations (low existing vaccination coverage), would re-introduce rabies cycles in such immunologically naïve dog populations (increased public health impact). Therefore, to eliminate canine-mediated rabies from South Africa, disease control and elimination efforts would ideally need to focus on both domestic dogs (e.g., regular parenteral vaccination) and sylvatic populations (oral rabies vaccines, ORVs) in the provinces where endemic cycles of sylvatic rabies are known to occur. Indeed, Bingham et al. (1999) tested the efficacy of the SAG-2 ORV in jackal populations in Zimbabwe and found them to be effective at achieving an adequate level of seroconversion in target populations [41]—confirming the feasibility of using ORVs in southern African countries.

## Data Availability

Publicly available datasets were analysed in this study. This data can be found here: https://www.ncbi.nlm.nih.gov/genbank/ (accessed 23 May 2022) according to Accession Numbers used in this study.

## Supplementary Materials

The following supporting information can be downloaded at: https://www.mdpi.com/article/10.3390/tropicalmed7060090/s1, Table S1: List of RABV sequences included in the phylogenetic analysis for both the partial N gene and G-L intergenic region.

## References

[1] ICTV International Committee on Taxonomy of Viruses (ICTV). https://talk.ictvonline.org/taxonomy/.

[2] WHO (2018). WHO Expert Consultation on Rabies: Third Report.

[3] Lembo T., Hampson K., Kaare M.T., Ernest E., Knobel D., Kazwala R.R., Haydon D.T., Cleaveland S. (2010). The Feasibility of Canine Rabies Elimination in Africa: Dispelling Doubts with Data. PLoS Negl. Trop. Dis..

[4] Vigilato M.A.N., Clavijo A., Knobl T., Silva H.M.T., Cosivi O., Schneider M.C., Leanes L.F., Belotto A.J., Espinal M.A. (2013). Progress towards Eliminating Canine Rabies: Policies and Perspectives from Latin America and the Caribbean. Philos. Trans. R. Soc. B Biol. Sci..

[5] Weyer J. (2015). Rabies in South Africa: Where Do We Stand in 2015?. South. African J. Infect. Dis..

[6] Hampson K., Coudeville L., Lembo T., Sambo M., Kieffer A., Attlan M., Barrat J., Blanton J.D., Briggs D.J., Cleaveland S. (2015). Estimating the Global Burden of Endemic Canine Rabies. PLoS Negl. Trop. Dis..

[7] Swanepoel R., Barnard B.J.H., Meredith C.D., Bishop G.C., Bruckner G.K., Foggin C.M., Hubschle O.J.B. (1993). Rabies in Southern Africa. Onderstepoort J. Vet. Res..

[8] Cohen C., Sartorius B., Sabeta C., Zulu G., Paweska J., Mogoswane M., Sutton C., Nel L.H., Swanepoel R., Leman P.A. (2007). Epidemiology and Molecular Virus Characterization of Reemerging Rabies, South Africa. Emerg. Infect. Dis..

[9] Sabeta C.T., Mansfield K.L., McElhinney L.M., Fooks A.R., Nel L.H. (2007). Molecular Epidemiology of Rabies in Bat-Eared Foxes (Otocyon Megalotis) in South Africa. Virus Res..

[10] Ngoepe C.E., Sabeta C., Nel L. (2009). The Spread of Canine Rabies into Free State Province of South Africa: A Molecular Epidemiological Characterization. Virus Res..

[11] Mkhize G.C., Ngoepe E.C., Du Plessis B.J.A., Reininghaus B., Sabeta C.T. (2010). Re-Emergence of Dog Rabies in Mpumalanga Province, South Africa. Vector-Borne Zoonotic Dis..

[12] Sabeta C.T., Weyer J., Geertsma P., Mohale D., Miyen J., Blumberg L.H., Leman P.A., Phahladira B., Shumba W., Walters J. (2013). Emergence of Rabies in the Gauteng Province, South Africa: 2010-2011. J. S. Afr. Vet. Assoc..

[13] Hergert M., Le Roux K., Nel L.H. (2018). Characteristics of Owned Dogs in Rabies Endemic KwaZulu-Natal Province, South Africa. BMC Vet. Res..

[14] Zulu G.C., Sabeta C.T., Nel L.H. (2009). Molecular Epidemiology of Rabies: Focus on Domestic Dogs (Canis Familiaris) and Black-Backed Jackals (Canis Mesomelas) from Northern South Africa. Virus Res..

[15] Kissi B., Tordo N., Bourhy H. (1995). Genetic Polymorphism in the Rabies Virus Nucleoprotein Gene. Virology.

[16] Nel L.H., Sabeta C.T., von Teichman B., Jaftha J.B., Rupprecht C.E., Bingham J. (2005). Mongoose Rabies in Southern Africa: A Re-Evaluation Based on Molecular Epidemiology. Virus Res..

[17] Bishop G.C., Durrheim D.N., Kloeck P.E., Godlonton J.D., Bingham J., Speare R., Advisory R. (2010). Guide for the Medical, Veterinary and Allied Professions.

[18] Van Niekerk H.N. (2010). The Cost of Predation on Small Livestock in South Africa by Medium-Sized Predators. Ph.D. Thesis.

[19] Cleaveland S. (1998). The Growing Problem of Rabies in Africa. Trans. R. Soc. Trop. Med. Hyg..

[20] King A.A., Fooks A.R., Aubert M., Wandeler A.I. (2004). Historical Perspective of Rabies in Europe and the Mediterranean Basin.

[21] Sabeta C.T., Mkhize G.C., Ngoepe E.C. (2011). An Evaluation of Dog Rabies Control in Limpopo Province (South Africa). Epidemiol. Infect..

[22] Coetzee P., Nel L.H. (2007). Emerging Epidemic Dog Rabies in Coastal South Africa: A Molecular Epidemiological Analysis. Virus Res..

[23] Shwiff S.A., Hatch B., Anderson A., Nel L.H., Leroux K., Stewart D., de Scally M., Govender P., Rupprecht C.E. (2016). Towards Canine Rabies Elimination in KwaZulu-Natal, South Africa: Assessment of Health Economic Data. Transbound. Emerg. Dis..

[24] Le Roux K., Kotze J., Perrett K. (2018). Elimination of Dog-Mediated Human Rabies: The Burden of Human Rabies in Africa. Rev Sci Tech Int Off Epizoot.

[25] Grewar J. Rabies Events in the Western Cape Province 2010 | Agriprobe. https://journals.co.za/doi/10.10520/EJC19017.

[26] Weyer J., Szmyd-Potapczuk A.V., Blumberg L.H., Leman P.A., Markotter W., Swanepoel R., Paweska J.T., Nel L.H. (2011). Epidemiology of Human Rabies in South Africa, 1983-2007. Virus Res..

[27] Markotter W., Kuzmin I., Rupprecht C.E., Randles J., Sabeta C.T., Wandeler A.I., Nel L.H. (2006). Isolation of Lagos Bat Virus from Water Mongoose. Emerg. Infect. Dis..

[28] Sacramento D., Bourhy H., Tordo N. (1991). PCR Technique as an Alternative Method for Diagnosis and Molecular Epidemiology of Rabies Virus. Mol. Cell. Probes.

[29] von Teichman B.F., Thomson G.R., Meredith C.D., Nel L.H. (1995). Molecular Epidemiology of Rabies Virus in South Africa: Evidence for Two Distinct Virus Groups. J. Gen. Virol..

[30] Cobb B.D., Clarkson J.M. (1994). A Simple Procedure for Optimising the Polymerase Chain Reaction (PCR) Using Modified Taguchi Methods. Nucleic Acids Res..

[31] Kumar S., Stecher G., Li M., Knyaz C., Tamura K. (2018). MEGA X: Molecular Evolutionary Genetics Analysis across Computing Platforms. Mol. Biol. Evol..

[32] Bouckaert R., Vaughan T.G., Barido-Sottani J., Duchêne S., Fourment M., Gavryushkina A., Heled J., Jones G., Kühnert D., De Maio N. (2019). BEAST 2.5: An Advanced Software Platform for Bayesian Evolutionary Analysis. PLOS Comput. Biol..

[33] Sabeta C.T., Marston D.A., McElhinney L.M., Horton D.L., Phahladira B.M.N., Fooks A.R. (2020). Rabies in the African Civet: An Incidental Host for Lyssaviruses?. Viruses.

[34] Brunker K., Marston D.A., Horton D.L., Cleaveland S., Fooks A.R., Kazwala R., Ngeleja C., Lembo T., Sambo M., Mtema Z.J. (2015). Elucidating the Phylodynamics of Endemic Rabies Virus in Eastern Africa Using Whole-Genome Sequencing. Virus Evol..

[35] Hayman D.T.S., Johnson N., Horton D.L., Hedge J., Wakeley P.R., Banyard A.C., Zhang S., Alhassan A., Fooks A.R. (2011). Evolutionary History of Rabies in Ghana. PLoS Negl. Trop. Dis..

[36] StatsSA North West Publication | Statistics South Africa. http://www.statssa.gov.za/?page_id=1854&PPN=Report-11-02-07&SCH=7908.

[37] Badenhorst C.G. (2014). The Economic Cost of Large Stock Predation in the North West Province of South Africa. Ph.D. Thesis.

[38] StatsSA Census of Commercial Agriculture. http://www.statssa.gov.za/publications/Report-11-02-01/CoCA2017FactSheets.pdf.

[39] Bingham J. (2005). Canine Rabies Ecology in Southern Africa. Emerg. Infect. Dis..

[40] LeRoux A., Balmforth Z., Mbatyoti O., Bizani M., Avenant N., Cavallini P., Do Linh San E., Child M., Roxburgh L., Do Linh San E., Raimondo D., Davies-Mostert H. (2016). A Conservation Assessment of Cynictis Penicillata. The Red List of Mammals of South Africa, Swaziland and Lesotho.

[41] Bingham J., Schumacher C.L., Hill F.W.G., Aubert A. (1999). Efficacy of SAG-2 Oral Rabies Vaccine in Two Species of Jackal (Canis Adustus and Canis Mesomelas). Vaccine.

