# Supplementary material for: Epidemiological Interface of Sylvatic and Dog Rabies in the North West Province of South Africa
Source: Trop Med Infect Dis. 2022 Jun 5;7(6):90. doi: 10.3390/tropicalmed7060090 (PMC9227852; doi:10.3390/tropicalmed7060090)
Supplement: Supplementary file 1 [file tropicalmed-07-00090-s001.zip › tropicalmed-1762595-supplementary.pdf]

Table S1: List of RABV sequences included in the phylogenetic analysis for both the partial N gene and G-L intergenic region.

Year Sampled	Sample Number	Species	Country	Province	Latitude	Longitude	Accession number (partial N gene)	Accession number (G-L gene region)
**1989**	399	Jackal	Botswana	Tsabong	-25.754	22.41838	AY330747	--
**1990**	420/90	Yellow mongoose	South Africa	North West	-27.1974	25.98311	FJ392383	AF079921
**1990**	m466	Yellow mongoose	South Africa	Free State	-27.374	26.61996	--	AF079922
**1991**	19671	African civet	Zimbabwe	Manicaland	-18.5279	32.12843	KY553266	AF304188
**1994**	22574	African civet	Zimbabwe	Mashonaland East	-18.6173	31.5736	KY553255	AF304183
**2003**	KZNdg03.453	Canine	South Africa	KwaZulu-Natal	-29.8579	31.0292	--	DQ841514
**2006**	696/06	Yellow mongoose	South Africa	Free State	-27.6504	27.23488	JQ692994	--
**2008**	UPV128	Canine	South Africa	KwaZulu-Natal	-29.8579	31.0292	JF747613	--
**2011**	11_185	Canine	South Africa	KwaZulu-Natal	-29.5093	30.19838	KJ744305	--
**2012**	556/12	Canine	South Africa	North West	-25.2775	27.21605	KT892003	MK103308
**2014**	889/14	African wild dog	South Africa	North West	-24.7435	26.25732	KT891999	--
**2015**	471/15	Canine	South Africa	North West	-27.2371	26.23514	MT454634	MT454634
**2015**	682/15	Canine	South Africa	North West	-24.7435	26.25732	MT454635	MT454635
**2015**	KZNbov15/261	Bovine	South Africa	KwaZulu-Natal	-29.4893	30.21665	--	KY681395

**2016**	516/16	Canine	South Africa	North West	-25.4261	27.2243	MT454636	MT454636
**2016**	635/16	Canine	South Africa	North West	-25.8026	27.87506	MT454639	MT454639
**2017**	**NWdog17/17**	Canine	South Africa	North West	-25.7905	27.2421	MW344002	MW343892
**2017**	**NWbov22/17**	Bovine	South Africa	North West	-26.9566	24.7284	MW344003	MW343893
**2017**	**NWdog31/17**	Canine	South Africa	North West	-25.1609	27.16296	MW344014	MW343904
**2017**	**NWbov57/17**	Bovine	South Africa	North West	-26.9566	24.7284	MW344005	MW343895
**2017**	**NWbov59/17**	Bovine	South Africa	North West	-27.1887	25.32931	MW344007	MW343897
**2017**	**NWbov62/17**	Bovine	South Africa	North West	-26.8648	24.79046	MW344015	MW343905
**2017**	**NWbov74/17**	Bovine	South Africa	North West	-26.9566	24.7284	MW344011	MW343901
**2017**	**NWbov126/17**	Bovine	South Africa	North West	-26.9566	24.7284	MW344016	MW343906
**2017**	**NWjac198/17**	Jackal	South Africa	North West	-26.6864	25.45907	MW344017	MW343907
**2017**	**NWbov331/17**	Bovine	South Africa	North West	-27.1887	25.32931	MW344018	MW343908
**2017**	**NWbov432/17**	Bovine	South Africa	North West	-26.1739	26.46947	MW343988	MW343878
**2017**	**NWbov435/17**	Bovine	South Africa	North West	-26.9566	24.7284	MW344004	MW343894
**2017**	**NWcap528/17**	Caprine	South Africa	North West	-27.5311	24.78659	MW343987	MW343877
**2017**	**NWovi583/17**	Ovine	South Africa	North West	-27.5311	24.78659	MW344006	MW343896
**2017**	**NWbov604/17**	Bovine	South Africa	North West	-26.9566	24.7284	MW343989	MW343879

**2017**	**NWcap608/17**	Caprine	South Africa	North West	-25.1334	26.86546	MW344008	MW343898
**2017**	**NWbov630/17**	Bovine	South Africa	North West	-26.125	23.7725	MW344009	MW343899
**2017**	**NWbbj666/17**	Black-backed jackal	South Africa	North West	-26.2802	25.10966	MW344010	MW343900
**2017**	**GPdog574/17**	Canine	South Africa	Gauteng	-25.4729	28.09919	MW344012	MW343902
**2017**	269/17	Canine	South Africa	North West	-26.9566	24.7284	MT454643	MT454643
**2017**	400/17	Canine	South Africa	North West	-26.9566	24.7284	MT454645	MT454645
**2017**	454/17	Black-backed jackal	South Africa	North West	-26.7167	27.1	MT454646	MT454646
**2017**	460/17	Black-backed jackal	South Africa	North West	-26.5961	24.17612	MT454647	MT454647
**2017**	474/17	Black-backed jackal	South Africa	North West	-27.5311	24.78659	MT454649	MT454649
**2017**	477/17	Bat-eared fox	South Africa	North West	-26.6181	25.65319	MT454650	MT454650
**2017**	480/17	Black-backed jackal	South Africa	North West	-27.1887	25.32931	MT454651	MT454651
**2017**	466/17	Black-backed jackal	South Africa	North West	-26.7167	27.1	MT454648	MT454648
**2017**	483/17	Black-backed jackal	South Africa	North West	-26.3138	26.89865	MT454652	MT454652
**2017**	502/17	Black-backed jackal	South Africa	North West	-26.7167	27.1	MT454653	MT454653

**2017**	503/17	Black-backed jackal	South Africa	North West	-26.7167	27.1	MT454654	MT454654
**2017**	**LPbov354/17**	Bovine	South Africa	Limpopo	-24.5917	27.41155	MW343945	MW343835
**2018**	**NWdog44/18**	Canine	South Africa	North West	-26.152	26.15968	MW343985	MW343875
**2018**	**NWbbj110/18**	Black-backed jackal	South Africa	North West	-27.1718	26.12699	MW343983	MW343873
**2018**	**NWdog121/18**	Canine	South Africa	North West	-26.4677	26.83939	MW343984	MW343874
**2018**	**NWbbj135/18**	Black-backed jackal	South Africa	North West	-26.9566	24.7284	MW343990	MW343880
**2018**	**NWbbj195/18**	Black-backed jackal	South Africa	North West	-26.2833	26.8	MW343986	MW343876
**2018**	**NWdog270/18**	Canine	South Africa	North West	-25.354	26.53009	MW344019	MW343909
**2018**	**NWdog293/18**	Canine	South Africa	North West	-25.1334	26.86546	MW344020	MW343910
**2018**	**NWbov299/18**	Bovine	South Africa	North West	-27.4377	25.13069	MW343991	MW343881
**2018**	**NWbbj343/18**	Black-backed jackal	South Africa	North West	-27.914	25.16111	MW343981	MW343871
**2018**	**NWbov382/18**	Bovine	South Africa	North West	-26.152	26.15968	MW343997	MW343887
**2018**	**NWbbj387/18**	Black-backed jackal	South Africa	North West	-26.8097	27.28492	MW343998	MW343888
**2018**	**NWdog391/18**	Canine	South Africa	North West	-26.8351	27.04304	MW343999	MW343889

**2018**	**NWdog405/18**	Canine	South Africa	North West	-25.8963	27.42684	MW344021	MW343911
**2018**	**NWdog420/18**	Canine	South Africa	North West	-25.605	27.91	MW344000	MW343890
**2018**	**NWovi429/18**	Ovine	South Africa	North West	-26.4748	27.06278	MW344001	MW343891
**2018**	**NWjac455/18**	Jackal	South Africa	North West	-26.152	26.15968	MW343978	MW343868
**2018**	**NWgen516/18**	Genet	South Africa	North West	-25.537	26.07512	MW344022	MW343912
**2018**	**NWbef103/18**	Bat-eared fox	South Africa	North West	-26.1944	24.92368	MW344013	MW343861
**2019**	**GPbov309/19**	Bovine	South Africa	Gauteng	-26.0858	27.77515	MW343994	MW343884
**2019**	**NWbov76/19**	Bovine	South Africa	North West	25.16111	24.17612	MW343969	MW343859
**2019**	**NWbbj96/19**	Black-backed jackal	South Africa	North West	-26.3138	26.89865	MW343970	MW343860
**2019**	**NWcap103/19**	Caprine	South Africa	North West	-26.8091	26.00538	MW343971	MW343861
**2019**	**NWbov109/19**	Bovine	South Africa	North West	-26.9566	24.7284	MW343972	MW343862
**2019**	**NWbov151/19**	Bovine	South Africa	North West	-25.1334	26.86546	MW343982	MW343872
**2019**	**NWdog169/19**	Canine	South Africa	North West	-26.8091	26.00538	MW343975	MW343865
**2019**	**NWaard171/19**	Aardwolf	South Africa	North West	-25.6676	27.24208	MW343976	MW343866
**2019**	**NWdog191/19**	Canine	South Africa	North West	-25.6676	27.24208	MW343973	MW343863
**2019**	**NWbbj219/19**	Black-backed jackal	South Africa	North West	-25.6676	27.24208	MW343974	MW343864

**2019**	**NWbbj248/19**	Black-backed jackal	South Africa	North West	-26.8521	26.66672	MW343977	MW343867
**2019**	**NWjac325/19**	Jackal	South Africa	North West	-25.6676	27.24208	MW343979	MW343869
**2019**	**NWbov331/19**	Bovine	South Africa	North West	-25.354	26.53009	MW343980	MW343870
**2019**	**NWbov379/19**	Bovine	South Africa	North West	-26.9333	25.41667	MW343992	MW343882
**2019**	**NWbov380/19**	Bovine	South Africa	North West	-27.2231	25.27706	MW343995	MW343885
**2019**	**NWbov428/19**	Bovine	South Africa	North West	-26.2	25.9	MW343996	MW343886

Sample numbers for sequences generated in this study are shown in bold.
